# Open access and medicinal chemistry

**DOI:** 10.1186/1752-153X-1-2

**Published:** 2007-02-19

**Authors:** Chris Swain

**Affiliations:** 1 Cambridge MedChem Consulting, Duxford, Cambridgeshire, CB2 4RN.

## Abstract

Chemistry Central is a new open access website for chemists publishing peer-reviewed research in chemistry from a range of open access journals. A new addition, Chemistry Central Journal, will cover all of chemistry and will be broken down into discipline-specific sections, and Im delighted that Medicinal Chemistry will be a key discipline in this new journal.

The open access model for scientific publication continues to gain support and is already well established in many areas of science and mathematics. BioMed Central [1] has been at the forefront of this movement and now has an impressive portfolio of over 60 journals [2]. In addition there are over 100 independent journals supported by BioMed Central [3]. The importance to the scientific community is underlined by the fact that Thomson Scientific (ISI) tracks many BioMed Central titles, and several have already achieved impressive Impact Factors [4]. In addition, all BioMed Central journal articles are tracked by Scopus [5] and Google Scholar [6]. Studies [7] suggest that open-access articles across a number of disciplines have a greater impact than those that are not freely available, and wider indexing might be expected to increase visibility and impact.

Chemistry Central [8] is a new open access website for chemists publishing peer-reviewed research in chemistry from a range of open access journals. A new addition, Chemistry Central Journal [9], will cover all of chemistry and will be broken down into discipline-specific sections, and I'm delighted that Medicinal Chemistry will be a key discipline in this new journal.

In many ways medicinal chemistry provides a bridge between biology and chemistry, identifying novel compounds that modulate a biological response and thusproviding the tools to investigate biological processes, subsequently optimizing those compounds to avoid undesired interactions with other biological systems. The challenge of bringing a novel drug molecule to market has increased considerably over the last two decades. To meet this challenge the medicinal chemist's toolbox has expanded over the years to include, high-throughput screening, computer-aided design, X-ray crystallography, target protein mutagenesis, combinatorial and automated chemistry, QSAR, chemical property calculations, computational data analysis, high-throughput ADME assays. All of which would be suitable topics for the new Medicinal Chemistry section of the Chemistry Central Journal.

Given the vast range of potential topics I thought I'd perhaps highlight two for detailed comment. The cost and time taken to develop drugs has increased enormously over the last three decades [10] with the current average time taken for discovery and development being 9-12 years and the costs predicted to top $1 billion. It is thus imperative that compounds brought forward do not fail in late stage development and the two features that have a major impact are pharmacokinetics and toxicity. In 1997 Lipinski published a key paper identifying molecular features that appear to be found in orally available drugs [11] the Lipinski "rule of five" has since become the yardstick by which potential development compounds are judged. It has also spawned an ongoing analysis of molecular properties and how they might be used to improve the drug discovery process from lead identification to drug development.

The Human Ether-a-go-go gene (HERG) encodes a potassium ion channel responsible for the repolarizing current in the cardiac action potential (the HERG gene is related to the Ether-a-go-go gene found in the Drosophila fly. When flies with mutations in this gene are anaesthetized with ether, their legs start to shake, like the dancing then popular at the Whisky A Go-Go nightclub in West Hollywood, California. Ether-a-go-go was named in the 1960s by William D. Kaplan). A number of drugs have been shown to bind to this channel causing decreased channel function and causing QT prolongation and fatal cardiac arrhythmias. In 2000 Mitcheson *et al *published a seminal paper in PNAS giving a molecular insight into how structurally unrelated molecules might block the ion channel [12]. This has lead to a number of predictive pharmacophore models or docking studies trying to eliminate this unwanted activity. Many of these studies would benefit from open access to larger data sets to enable refinement of the models and this journal could potentially provide such information.

Online publishing offers many practical advantages beyond electronic handling of manuscripts right up to the point of publication. Online formats aid the support for the use of color to create high quality figures, graphs, and color images. In addition many technologies not available in print-only formats can be explored such as hyperlinking, video files, and interactive graphics and molecular superimpositions. Figures will be able to be submitted in ChemDraw (.CDX) or ISIS/Draw (.TGF) file formats. All articles will be deposited in PubMed Central [13], and will therefore be automatically linked into PubChem [14], based on the chemical substances that they mention. Additional information such as experimental or spectroscopic data assisting the reader can, and will, be provided liberally and could of course be linked electronically to structures within the document. The ready availability of supporting data should make this an invaluable data store for structure activity information for future work.
